# Consumption of *Anacardium occidentale* L. (Cashew Nuts) Inhibits Oxidative Stress through Modulation of the Nrf2/HO−1 and NF-kB Pathways

**DOI:** 10.3390/molecules25194426

**Published:** 2020-09-26

**Authors:** Roberta Fusco, Marika Cordaro, Rosalba Siracusa, Alessio Filippo Peritore, Enrico Gugliandolo, Tiziana Genovese, Ramona D’Amico, Rosalia Crupi, Antonella Smeriglio, Giuseppina Mandalari, Daniela Impellizzeri, Salvatore Cuzzocrea, Rosanna Di Paola

**Affiliations:** 1 Department of Chemical, Biological, Pharmaceutical and Environmental Sciences, University of Messina, 98166 Messina, Italy; rfusco@unime.it (R.F.); rsiracusa@unime.it (R.S.); aperitore@unime.it (A.F.P.); egugliandolo@unime.it (E.G.); tgenovese@unime.it (T.G.); rdamico@unime.it (R.D.); asmeriglio@unime.it (A.S.); gmandalari@unime.it (G.M.); dipaolar@unime.it (R.D.P.); 2 Department of Biomedical, Dental and Morphological and Functional Imaging University of Messina, Via Consolare Valeria, 98125 Messina, Italy; cordarom@unime.it; 3 Department of Veterinary Sciences, University of Messina, 98168 Messina, Italy; rcrupi@unime.it; 4 Department of Pharmacological and Physiological Science, Saint Louis University School of Medicine, Saint Louis, MO 63104, USA

**Keywords:** medicinal plants, cashew nuts, antioxidant

## Abstract

Ischemia/reperfusion injury is a severe disorder associated with a high mortality. Several antioxidant and pharmacological properties of cashew nuts (*Anacardium occidentale* L.) and its metabolites from different countries have recently been described. It is a medicinal plant with important therapeutic effects. This study aimed to verify the effect of an oral administration of cashew nuts in a rat model of ischemia/reperfusion (I/R). Adult male rats were subjected to intestinal I/R injury by clamping the superior mesenteric artery for 30 min and then allowing animals to 1 h of reperfusion. Rats subjected to I/R of the gut showed a significant increase in different biochemical markers. In particular, we evaluated lipid peroxidation, tissue myeloperoxidase activity, protein carbonyl content, reactive oxygen species generation and decreased antioxidant enzyme activities. Western blot analysis showed the activation of the NRF2 and NF-kB pathways. Increased immunoreactivity to nitrotyrosine, PARP, P-selectin, and ICAM-1 was observed in the ileum of rats subjected to I/R. Administration of cashew nuts (100 mg/kg) significantly reduced the mortality rate, the fall in arterial blood pressure, and oxidative stress and restored the antioxidant enzyme activities by a mechanism involving both NRF2 and NF-kB pathways. Cashew nuts treatments reduced cytokines plasma levels, nitrotyrosine, and PARP expression as well as adhesion molecules expressions. Additionally, cashew nuts decreased the intestinal barrier dysfunction and mucosal damage, the translocation of toxins and bacteria, which leads to systemic inflammation and associated organs injuries in particular of liver and kidney. Our study demonstrates that cashew nuts administration exerts antioxidant and pharmacological protective effects in superior mesenteric artery occlusion–reperfusion shock.

## 1. Introduction

Intestinal ischemia/reperfusion (I/R) is a severe event induced by trauma, mesenteric ischemia, sepsis shock, and surgical procedure. It caused increased intestinal permeability and leads to the release of abundant bacteria and their antigens into mesenteric lymph and circulation producing multiple organ dysfunction syndrome (MODS) and systemic response syndrome (SIRS) [1,2]. Although several evidences characterize the pathogenesis of intestinal injury and the related organ dysfunction, the underlying mechanisms still remain the subject of examination, and no effective therapeutic tools control or prevent the process. Oxidative stress has a critical part during intestinal I/R injury. Reactive oxygen species (ROS) are overproduced in injured cells and tissues following I/R injury, triggering to necrosis or apoptosis, amplifying pro-inflammatory response, and destroying the gut mucosal barrier [3]. The endothelial dysfunction produced by the injury recruits polymorphonuclear leukocytes to the lesion site involving a complex system of adhesion molecules [4,5]. Neutrophil activation in turn activates a large production of superoxide anions (O2−) [4], lipid peroxidation and oxidation [6], and DNA single-strand damage [7]. Moreover, O2− interacts with NO forming peroxynitrite (ONOO−), a potent proinflammatory and cytotoxic molecule [8]. Nuclear factor E2-related factor 2 (Nrf2) pathway is described to have a critical role in the pathogenesis of I/R injury [9]. It and its target genes are considered as multiple-organ protector thanks to the cytoprotective and antioxidative functions [10]. In particular, Nrf2 increased expression has been described to protect epithelial barrier function and gut inflammation [11,12]. Intake of fresh fruit, polyphenol-rich food, and vegetables is important to fight the oxidative effect of radical oxygen species (ROS) [13]. Nowadays, medicinal plants are widely used to treat different medical conditions. Numerous preclinical and clinical reports described the antimicrobial and antioxidant properties of plant compounds and their by-products [14]. *Anacardium occidentale* L. is a medicinal plant with important therapeutic effects [15]. In particular, for the different parts of the plant, such as stem, flowers, leaves, and fruits, different pharmacological functions have been described [16]. Additionally, the secondary metabolites included in the parts of the plants such as carotenoids, vitamin C and polyphenols, display great antioxidant effects. Among them, the most representative compounds were p-hydroxybenzoic, protocatechuic and gallic acids as well as aglycone and glycosylated flavonoids such as quercetin, myricetin, kaempferol and naringenin [17]. However, it is important to underline that tree from different countries has different chemical compositions. In this study for the first time, we evaluated the chemical composition and the antioxidant effects of the cashew nuts (*Anacardium occidentale L.*). Recent studies showed that the cashew nuts have cardioprotective properties [18], have cholesterol-lessening effect [19], and decrease the inflammatory mediators [20]. In particular, cashew nuts have elevated nutritional importance: they can modulate risk of cardiovascular issues, metabolic syndrome [21], chronic inflammatory bowel disorders [15], and painful degenerative joint disease [22]. The positive effects of natural antioxidant compound such as bergamot juice [23], glycyrrhizin [24], green tea [25], *Himanthalia elongata* [26], thymoquinone, and melatonin [27] have been also described. Accordingly, our study evaluated the effect of the oral treatment of cashew nuts from Coted’Ivoire in an experimental rat model of gut ischemia/reperfusion injury.

## 2. Results

### 2.1. Composition of Cashew Kernel Samples

Table 1 reports the nutritional profile of cashew kernel samples from Ivory Coast (Africa). In agreement with the chemical composition of West Africa cashew [15], cashews contain a high proportion of lipid (44.19%), followed by carbohydrate (35.43%) expressed as sum of soluble sugars and fiber and protein (22.46%). The total phenolic content, expressed as mg/100 g of sample, was higher (80.01) compared to what was reported in our previous work for West Africa cashews (69.64) [22].

### 2.2. Cashew Nuts Reduce Mortality, Fall of Arterial Blood Pressure, and Histological Changes Induced by Ischemia/Reperfusion Injury

**Figure 1:**
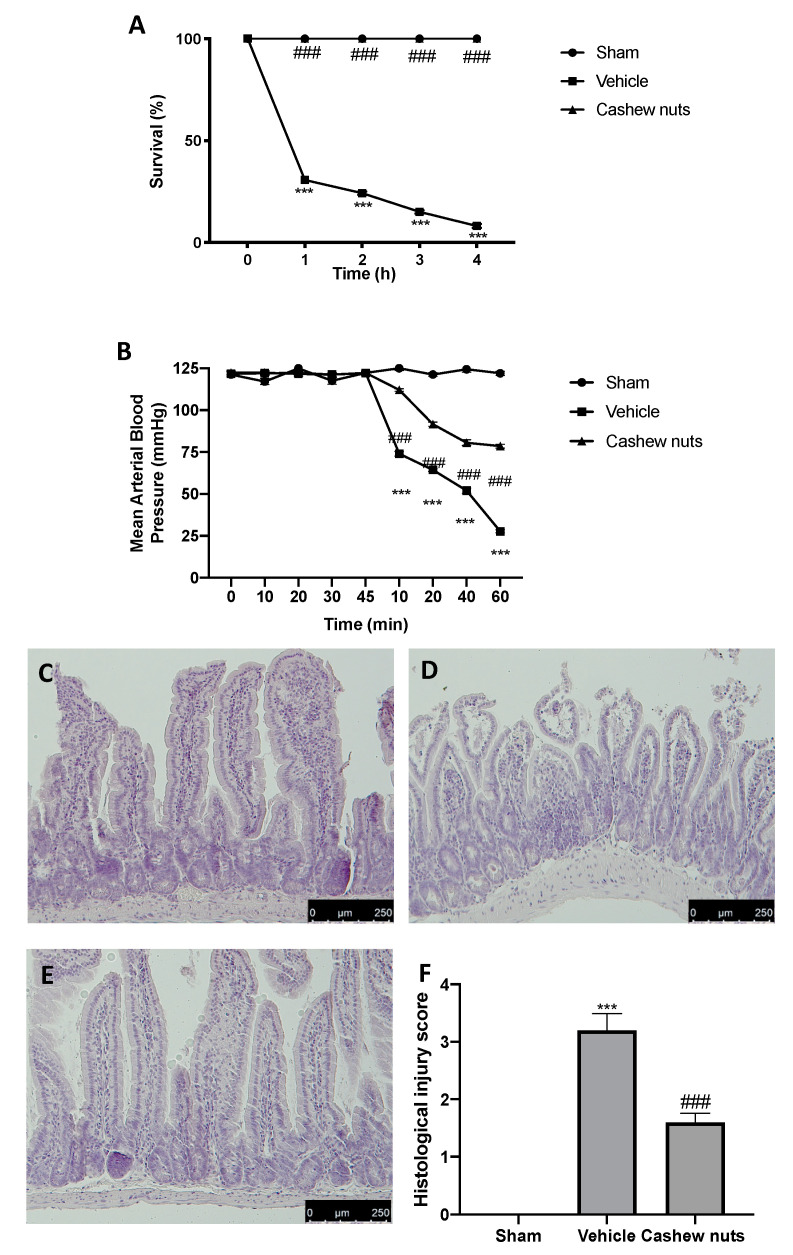
Effect of cashew nuts on ischemia/reperfusion (I/R) shock-induced mortality, mean arterial blood pressure, and intestine damage: survival % (**A**), mean arterial blood pressure (**B**), H&E staining: sham (**C**), vehicle (**D**), cashew nuts (**E**), histological injury score (**F**). Results were analyzed by the *t*-test. A *p*-value < 0.05 was considered significant. * *p* < 0.05 vs. sham, # *p* < 0.05 vs. vehicle, ** *p* < 0.01 vs. sham, ## *p* < 0.01 vs. vehicle, *** *p* < 0.001 vs. sham, ### *p* < 0.001 vs. vehicle.

Superior mesenteric artery occlusion caused an intense shock state producing 100% mortality in the vehicle-treated animals at the end of the 4 h reperfusion-period (Figure 1A), while all sham animals survived to the 4 h observation period. Moreover, 1 h after reperfusion, vehicle-treated animals showed a reduction of mean arterial blood pressure (Figure 1B). Cashew nuts administration significantly reduced the lethality induced by I/R (Figure 1A) and the fall in blood pressure observed after reperfusion (Figure 1B). Histological analysis was performed on ileum tissue of all animals of different groups. Tissue harvested from sham animals did not show any pathological feature (Figure 1C,F). Ileum sections collected from vehicle-treated animals showed alteration of the villi tips, diffuse swelling, and inflammatory cells infiltrate in the submucosa (Figure 1D,F). Melatonin (10 mg/kg IP), employed as positive control, showed protective effects on ileum tissue (Figure S1). Cashew nuts treatment significantly decreased ileum damage induced by I/R (Figure 1E,F).

### 2.3. Cashew Nuts Reduced Adhesion Molecules Expressions and Neutrophils Accumulation 

**Figure 2:**
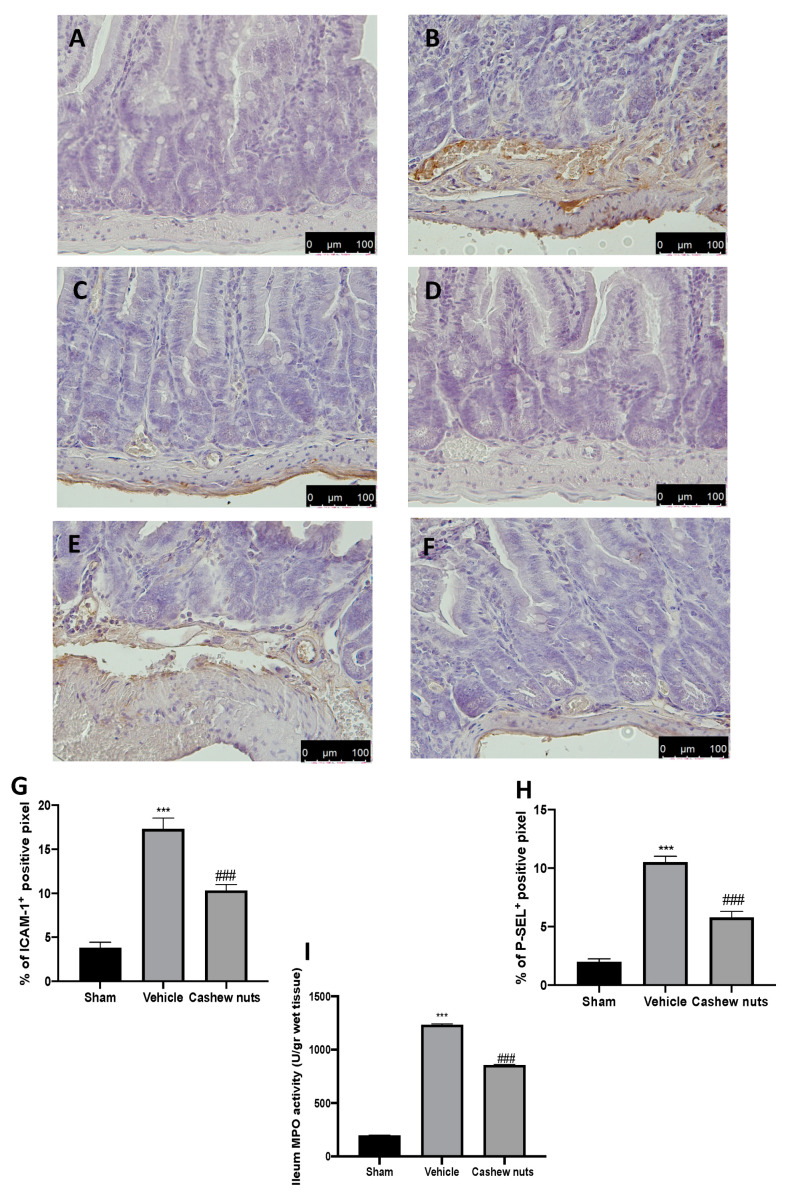
Effect of cashew nuts on, ICAM-1 (intercellular adhesion molecule), and P-selectin expressions and myeloperoxidase (MPO) activity induced by I/R shock: immunohistochemical analysis of ICAM−1 expression: sham (**A**), vehicle (**B**), cashew nuts (**C**), % of positive pixels (**G**), immunohistochemical analysis of P-selectin expression: sham (**D**), vehicle (**E**), cashew nuts (**F**), % of positive pixels (**H**), and MPO activity (**I**). Results were analyzed by the *t*-test. A *p*-value < 0.05 was considered significant. * *p* < 0.05 vs. sham, # *p* < 0.05 vs. vehicle, ** *p* < 0.01 vs. sham, ## *p* < 0.01 vs. vehicle, *** *p* < 0.001 vs. sham, ### *p* < 0.001 vs. vehicle.

The intestinal expression of the adhesion molecules ICAM-1 and P-selectin was investigated. Immunohistochemical analysis revealed increased expression of both molecules in tissues harvested from vehicle-treated rats (Figure 2B,G,E,H, respectively) as compared to the sham tissues (Figure 2A,G,D,H, respectively). Cashew nuts administration was able to reduce both ICAM−1 (Figure 2C,G) and P-selectin (Figure 2F,H) expression in ileum tissues. Vehicle-treated rats also showed increased myeloperoxidase activity in inflamed ileum, which was reduced by cashew nuts treatment (Figure 2I).

### 2.4. Cashew Nuts Enhances the Antioxidant/Oxidant Balance during Ischemia/Reperfusion Injury

**Figure 3:**
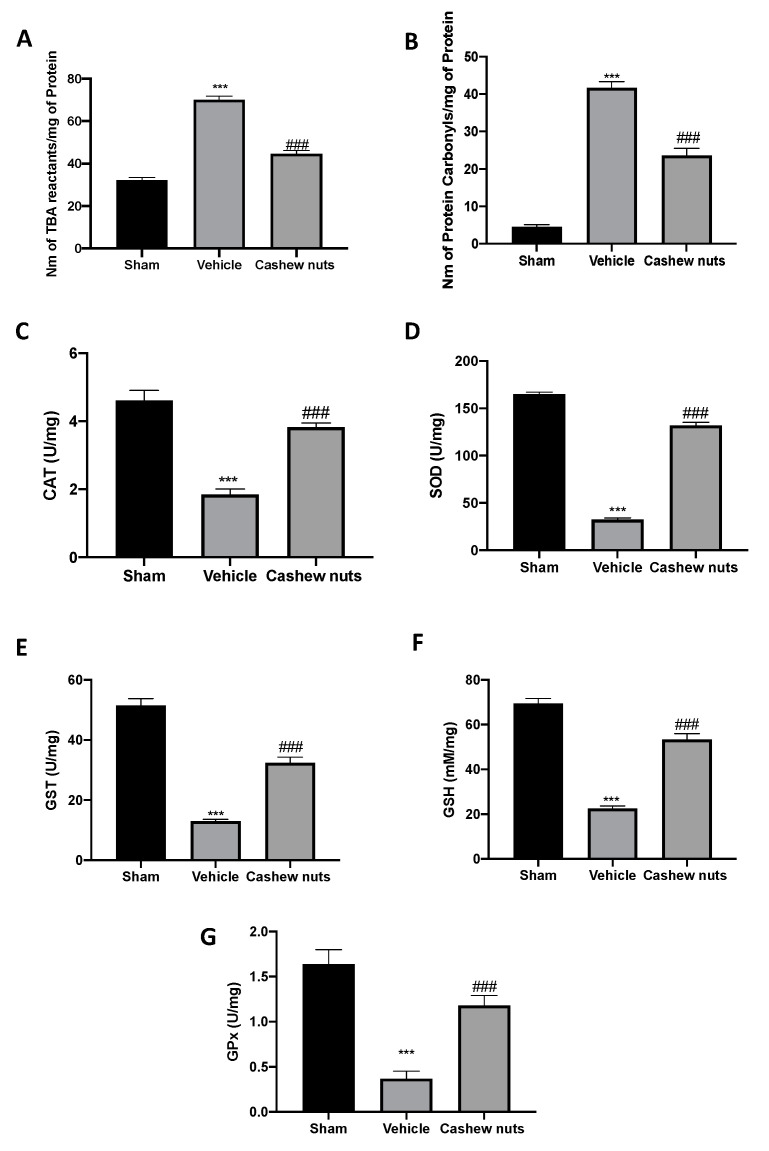
Effect of cashew nuts on oxidative stress and antioxidant enzymes modifications induced by I/R shock: lipid peroxidation (**A**), protein carbonyl content (**B**), catalase (CAT) (**C**), superoxide dismutase (SOD) (**D**), glutathione-S-transferase (GST) (**E**), glutathione (GSH) (**F**), and GPx (Glutathione peroxidase) (**G**). Results were analyzed by the *t*-test. A *p*-value < 0.05 was considered significant. * *p* < 0.05 vs. sham, # *p* < 0.05 vs. vehicle, ** *p* < 0.01 vs. sham, ## *p* < 0.01 vs. vehicle, *** *p* < 0.001 vs. sham, ### *p* < 0.001 vs. vehicle.

Vehicle-treated rats displayed a significant rise in the lipid peroxide (Figure 3A) and PCC (Figure 3B). Meanwhile, cashew nuts administration importantly restored the lipid peroxidation levels and PCC to the sham group levels (Figure 3A,B, respectively). I/R in vehicle-treated rats also decreased the antioxidant enzyme activities of CAT, SOD, GST, GPx, and GSH as compared to sham animals. Cashew nuts treatment restored the antioxidant status modified by I/R injury (Figure 3C–G, respectively).

### 2.5. Cashew Nuts Downregulates Nitrotyrosine and PARP Expression during Ischemia/Reperfusion Injury

**Figure 4:**
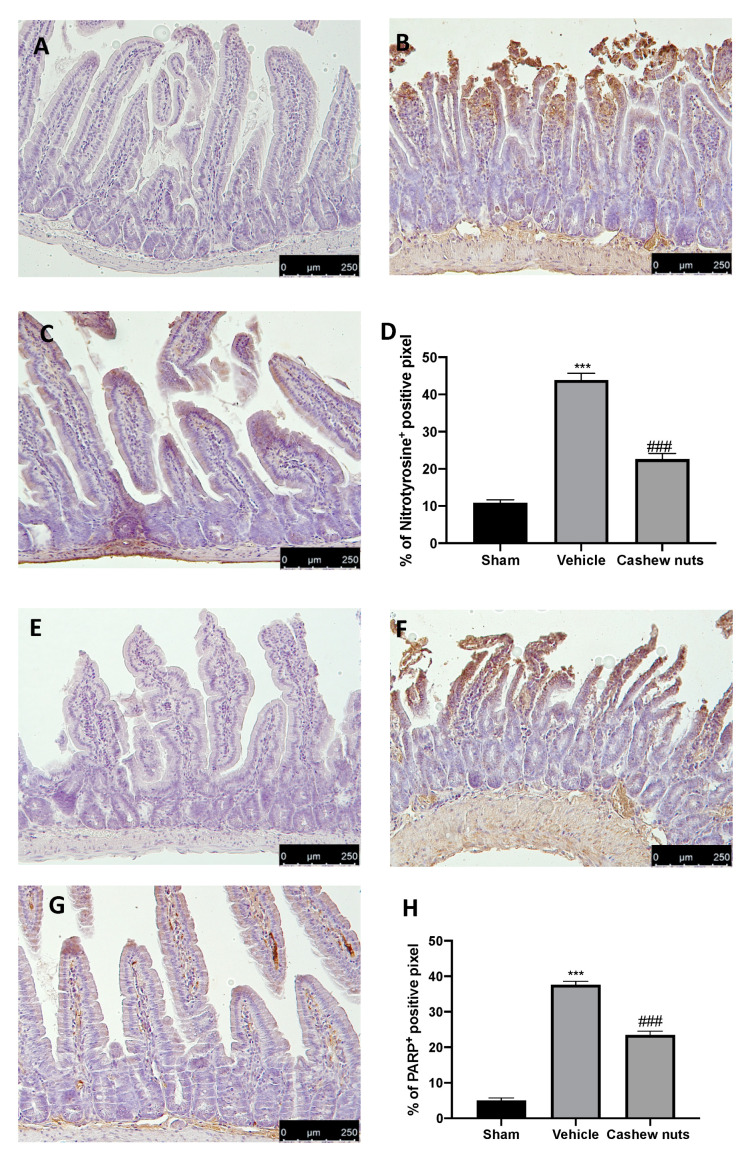
Effect of cashew nuts on nitrotyrosine and poly(ADP-ribose polymerase) (PARP) expression induced by I/R shock: immunohistochemical analysis of nitrotyrosine expression: sham (**A**), vehicle (**B**), cashew nuts (**C**), % of positive pixels (**D**), immunohistochemical analysis of PARP expression: sham (**E**), vehicle (**F**), cashew nuts (**G**), and % of positive pixels (**H**). Results were analyzed by the *t*-test. A *p*-value < 0.05 was considered significant. * *p* < 0.05 vs. sham, # *p* < 0.05 vs. vehicle, ** *p* < 0.01 vs. sham, ## *p* < 0.01 vs. vehicle, *** *p* < 0.001 vs. sham, ### *p* < 0.001 vs. vehicle.

**Figure 5:**
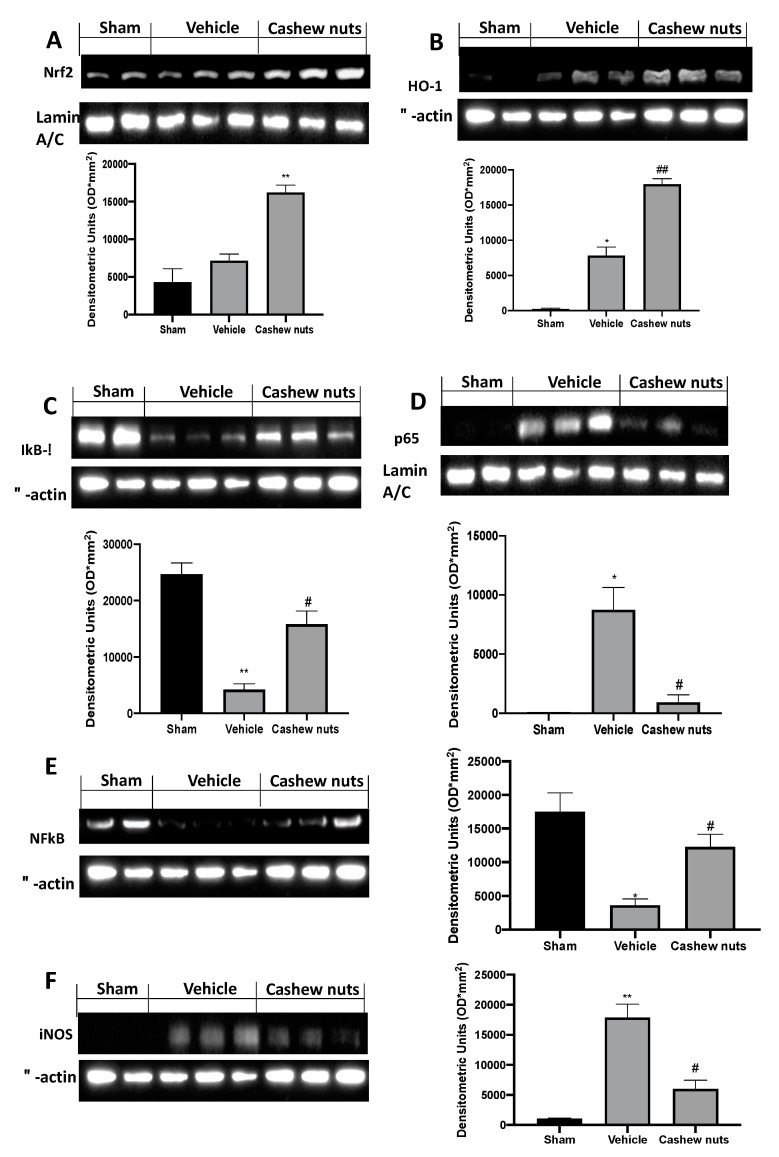
Effect of cashew nuts on nuclear factor E2-related factor 2 (Nrf2), heme oxygenase−1(HO−1), Ikb-α (nuclear factor of kappa light polypeptide gene enhancer in B-cells inhibitor, alpha), NF-kB (nuclear factor kappa-light-chain-enhancer of activated B cells), and inducible nitric oxide synthase (iNOS) expression induced by I/R shock: Western blot analysis of: Nrf2 (**A**), HO−1 (**B**), Ikb-α (**C**), nuclear NF-kB (**D**), cytoplasmic NF-kB (**E**), and iNOS (**F**). Results were analyzed by the *t*-test. A *p*-value < 0.05 was considered significant. * *p* < 0.05 vs. sham, # *p* < 0.05 vs. vehicle, ** *p* < 0.01 vs. sham, ## *p* < 0.01 vs. vehicle, *** *p* < 0.001 vs. sham, ### *p* < 0.001 vs. vehicle.

Tissue sections collected from vehicle-treated rats displayed positive staining for nitrotyrosine (Figure 4B,D) as compared to tissues from sham animals (Figure 4A,D). Moreover, a significant increase in PARP expression was detected in ileum tissue of vehicle-treated rats (Figure 5F,H) as compared to sham tissues (Figure 4E,H). Cashew nuts reduced nitrotyrosine and PARP expression in tissue collected from treated animals (Figure 4C,D,G,H, respectively).

### 2.6. Cashew Nuts Modulates Nrf2 and NF-kB Pathways during Ischemia/Reperfusion Injury

Western blot analysis of tissue sections collected from sham rats displayed basal Nrf2 expression, whereas in vehicle-treated rats, it was increased. Cashew nuts administration increased the expression of this transcription factor (Figure 5A). The same trend was observed for the HO−1 expression (Figure 5B): vehicle-treated rats showed increased HO-1 levels, as compared to the sham rats. Western blot analysis showed increased HO−1 in cashew nuts administered rats. We also evaluated the Ikb-α/NF-kB expression. I/R injury reduced Ikb-α (Figure 5C) and NF-kB expression in cytosol (Figure 5E), inducing increased NF-kB expression in the nucleus (Figure 5D). Cashew nuts restored Ikb-α expression in cytosol (Figure 5C) and reduced NF-kB levels into the nucleus (Figure 5D). Cashew nuts administration was able to reduce iNOS expression, which was upregulated in vehicle group as compared to tissues from sham animals (Figure 5E).

### 2.7. Cashew Nuts Modulates Intestinal Permeability, Bacterial Translocation, Cytokines Plasma Levels, and Renal and Hepatic Injuries during Ischemia/Reperfusion Injury

**Figure 6:**
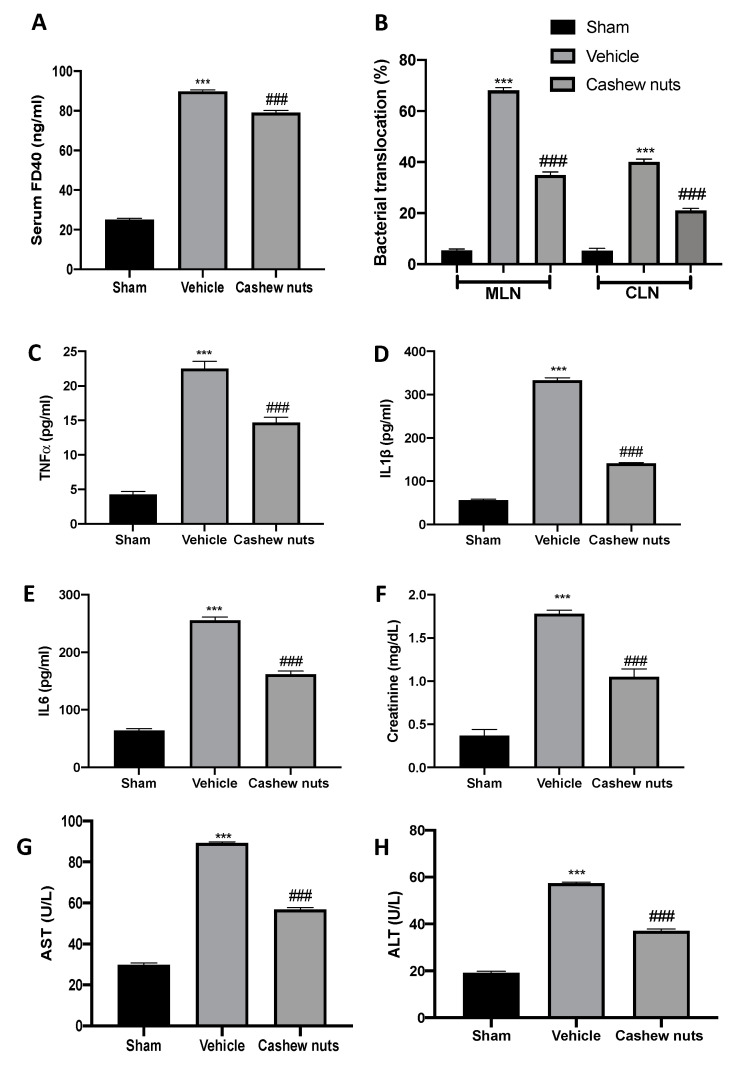
Effect of cashew nuts on intestinal barrier function, bacterial migration, and TNF-α (tumor necrosis factor alpha), Interleukin 6 (IL6), IL−1β, creatinine, glutamic oxaloacetic transaminase (AST), and alanine transaminase (ALT) levels induced by I/R shock: serum fluorescein isothiocyanate (FITC)-dextran (FD−40) levels (**A**), bacterial translocation (**B**), TNF-α (**C**), IL−1β (**D**), IL6 (**E**), creatinine (**F**), AST (**G**), and ALT levels (**H**). Results were analyzed by the *t*-test. A *p*-value < 0.05 was considered significant. * *p* < 0.05 vs. sham, # *p* < 0.05 vs. vehicle, ** *p* < 0.01 vs. sham, ## *p* < 0.01 vs. vehicle, *** *p* < 0.001 vs. sham, ###*p* < 0.001 vs. vehicle.

I/R injury augmented intestinal permeability increasing serum FD−40 as compared to the sham animals, while cashew nuts administration reduced serum FD−40 levels (Figure 6A). Additionally, vehicle-treated rats displayed increased bacterial translocation to MLN and CLN as compared to the sham rats. Cashew nuts oral treatment reduced bacterial translocation to different organs during I/R injury (Figure 6B). I/R injury increased TNF-α, IL6, and IL−1β plasma levels (Figure 6C–E). Cashew nuts administration significantly reduced cytokines plasma levels (Figure 6C–E). In addition, cashew nuts decreased the creatinine (Figure 6F), AST (Figure 6G), and ALT (Figure 6H) expression following intestinal I/R injury.

## 3. Discussion

Superior mesenteric artery occlusion leads to intestinal ischemia, acute renal failure, necrosis of legs, and multiple organ failure resulting in death. Several experimental animal models are used to understand the pathophysiologic mechanisms. Among them, I/R injury is a severe form of circulatory shock. This type of shock is characterized by an important fall in arterial blood pressure, which leads to release of hydrolases, hemoconcentration, enhanced proteolysis, production of cardiotoxic substances, and fatal outcome [28]. Furthermore, intestinal I/R injury induces systemic inflammation and distant organs injuries, including kidney and liver. Reactive oxygen species overexpression is one of the most-well described mechanisms occurring after an ischemic insult. In particular, ROS developed by hypoxia or reoxygenation interacts with signal transducers [29], leading to DNA oxidation, liposomal membrane lipid peroxidation, and enzyme proteins denaturation. Therapeutic tools able to manage ROS generation could represent important intervention for intestinal ischemia driven by oxidative stress. Well-known antioxidants such as quercetin, curcumin, and vitamin C protect against I/R injury [30,31], proposing that natural sources of antioxidants may be useful to treat this issue. Previous studies from our laboratory showed the antioxidant properties of cashew nuts in experimental model of colitis and osteoarthritis [15,22]. In particular, these studies showed cashew nuts’ ability to alleviate the clinical signs, pain perception, histological damage, and molecular mediators involved in these diseases. In this study, we investigated the effects of cashew nuts from Coted’Ivoire on reducing intestinal I/R injury, including renal and hepatic injury, and the possible pathway involved. Intestinal I/R injury is characterized by damaged villus surface and polymorphonuclear leukocytes recruitment at the lesion site [24]. MPO activity has been found increased in the intestinal I/R injury group, while cashew nuts administration reduced neutrophils activity. Moreover, endothelial–leukocyte interaction is mediated by adhesion molecules, such as ICAM−1 and P-selectin, which have been found upregulated in animals subjected to intestinal I/R injury. We demonstrate, here, that cashew nuts treatments significantly reduced positive staining of adhesion molecules. Free radicals mediate and amplify I/R shock injury. One of the main consequences of free radical generation is lipid peroxidation. The degree of lipid peroxidation is indicated by the formation of toxic aldehydes like MDA, which is an indicator of oxidative damage in tissue [25,32,33]. In this model of intestinal I/R injury, cashew nuts administration was able to decrease lipid peroxidation, ROS generation, and protein carbonyl content. Several works showed the decrease in CAT and SOD levels and GST, GPx, and GSH activities in both patients and experimental models, confirming the role of oxidative stress in intestinal ischemia pathogenesis [34]. These enzymes help cells to repair the cellular membranes damaged by ROS. Among these antioxidant enzymes, SOD is the primary enzyme of gastric mucosa, localized in mitochondria, cytosol, and extracellular matrix [35]. SOD converts the excess of superoxide anions in H_2_O_2_, which is then removed by CAT and GPx [36], and counteracts the gastrointestinal injury induced by I/R [37]. Cashew nuts’ oral treatment was able to restore the antioxidant enzymes activity in I/R injured animals. Parallel to the decrease of the lipid peroxidation, there is also the reduction of nitrotyrosine immunoreactivity. Nitrotyrosine formation was originally considered as a peroxynitrite marker [38]. It is a reactive oxidant and produce nitration of protein tyrosine residues and free tyrosine. Later some evidences propose that other reactions induce tyrosine nitration [39]. Increased nitrotyrosine expression is considered as an indicator of the increased nitrosative stress rather than a peroxynitrite marker. Moreover, peroxynitrite and ROS cause DNA strand breaks activating DNA repair mechanisms. Several evidences describe the activation of the nuclear enzyme PARS during intestinal I/R injury [40]. We showed that treatment with cashew nuts reduced nitrotyrosine and PARP expression. Furthermore, oxidative stress stimulates the activation of the redox-sensitive transcription factors, which in turn manages the expression of pro-inflammatory mediators. The Nrf2 and NF-kB pathway are closely related in the oxidative stress [41] and inflammatory [42] answers, respectively. They are transcription factors usually localized in the cytosol and regulated by Kelch-like ECH-associated protein 1 (Keap1) and IκBα inhibitor, respectively. ROS generation induces Nrf2 and NF-kB translocations into the nucleus where they modulate the transcription of multiple target genes. In particular, NRF2 regulates the expressions of detoxification enzymes, while NF-kB induces the transcription of pro-inflammatory mediators. Cashew nuts administration counteracts the propagation of the oxidative stress and inflammation by increasing NRF2 and reducing NF-kB translocation into the nucleus. Nrf2 activation enhances the antioxidant capacity and protects against oxidative damage improving the expression of antioxidant response genes [43], such as heme oxygenase−1 (HO−1). Consistently with the enhanced expression of NRF2, cashew nuts administration was able to increase HO−1 expression.

Inflammatory answer is an important factor to the exacerbation of I/R injury and uncontrolled inflammation and advanced tissue damage [44]. I/R injury induces to the translocation of toxins and bacteria, leading to systemic inflammation and in the occurrence of MODS and SIRS. Regarding the distant organs injuries induced by I/R, occlusion of the superior mesenteric artery induces local intestinal and multiple organs damage, such as liver and kidney [45]. As far as we know, we investigated for the first time, the protective effect of cashew nuts on intestinal I/R-associated organs injuries. Our work also showed increased plasma levels of pro-inflammatory cytokines, particularly TNF-α, IL6, and IL−1β, after intestinal I/R [46,47]. In response to growth factors and cytokines overexpression injured tissues produce the inducible form of NOS (iNOS). The large amount of NO produced by iNOS contributes to the tissue damage induced by the I/R injury [48]. Cashew nuts administration reduced iNOS expression and TNF-α, IL6, and IL−1β plasma levels. Additionally, cashew nuts administration reduced renal and hepatic injury during I/R, as showed by serum levels of creatinine, AST, and ALT.

In conclusion, we reported that cashew nuts administration displays beneficial action for the treatment of I/R injury. The biochemical assays revealed that cashew nuts were able to decrease lipid peroxidation, tissue myeloperoxidase activity, protein carbonyl content, and reactive oxygen species generation; restored antioxidant enzyme activities; and decreased secretion of pro-inflammatory cytokines. Western blot analysis showed modulations of transcription factors. Moreover, the histological data showed reduced tissue damage, neutrophil infiltration, and adhesion molecule expression. Additionally, cashew nuts would be useful for treatment of the systemic inflammatory response to I/R injury. Thus, treatment with cashew nuts is very promising and could be investigated in the prevention of the ischemic diseases.

## 4. Materials and Methods

### 4.1. Materials

Cashew kernel samples (*Anacardium occidentale* L.) obtained from Ivory Coast (Africa) were used. All chemicals were purchased from Sigma-Aldrich, and stock solutions were prepared in saline (0.9% NaCl; Baxter, Milan, Italy). Solvents were obtained from Merck (Darmstadt, Germany).

### 4.2. Characterization of Cashew Samples

#### 4.2.1. Moisture Determination

The moisture content of cashew samples (10 g) was estimated according to the Association of Official Analytical Chemists (AOAC) Official Method 925.40 (1995) [49]. The results of moisture content are expressed as percentage of fresh weight.

#### 4.2.2. Total Protein Determination

Total nitrogen in cashew samples (0.1 g) was determined by micro-Kjeldahl according to the AOAC method 950.48 (1995) [49]. Protein content was calculated as N × 6.25 and expressed as a percentage fresh weight.

#### 4.2.3. Lipid Content Determination

Lipid extraction of cashew samples (10 g) was performed using a Soxhlet apparatus according to the AOAC Official Method 948.22 (1995) [49]. The results are expressed as percentage of fresh weight.

#### 4.2.4. Dietary Fiber Determination

Dietary fiber content of cashews (1 g) was determined according to the AOAC Official Method 985.29 (1997) [50]. Results are expressed as percentage of fresh weight.

#### 4.2.5. Total Soluble Sugars

The soluble sugar content of cashew samples was detected according to the method developed by Dubois et al. (1956) and modified by Agrawal et al. (2015) [51,52].

#### 4.2.6. Ash Determination

Ash determination of cashew samples (5 g) was carried out according to the AOAC Official Method 923.03 (1995) [49]. Ash content was expressed as percentage of fresh weight.

#### 4.2.7. Polyphenols Extraction

A cashew extract was prepared as described by [53] with some modifications. Briefly, 10 g of cashews was extracted three times with 20 mL of n-hexane (Merck, Darmstadt, Germany) for 6 h under agitation in order to discard the fat. After filtration, the residue was mixed with 100 mL of methanol/HCl (Merck, Darmstadt, Germany) 0.1% (*v/v*), sonicated for 15 min, and centrifuged (5000× *g*, 10 min, 4 °C). The extraction was repeated twice, followed by concentration of the methanol fraction in a rotary evaporator. The residue was then dissolved in Milli-Q water (40 mL) and extracted four times with 40 mL of ethyl acetate (Merck, Darmstadt, Germany). After combining the organic phases, Na_2_SO_4_ was used for 20 min to dry them.

#### 4.2.8. Total Phenols (TP) Determination

The total phenolic content was determined according to the Folin–Ciocalteu method described by [24] with some modifications. Briefly, 50 µL of sample was added to Folin–Ciocalteu reagent (500 µL) followed by deionized water (450 µL). After 3 min, 500 µL, 10% *w/v* of sodium carbonate (Sigma-Aldrich, Milan, Italy) was added and samples were left in the dark at room temperature for 1 h, vortexing every 10 min. Absorbance was measured at 785 nm using a UV–Vis spectrophotometer (Shimadzu UV-1601, Kyoto, Japan). Results are expressed as milligram of gallic acid equivalents (GAE)/100 g of fresh weight.

### 4.3. Animals

Male rats (Sprague–Dawley (200–230 g, Envigo, Milan, Italy)) were employed in this research. Animals were located in the University of Messina, and all experiments were conducted there. The University of Messina Review Board for animal care (OPBA) approved the study. All animal experiments agree with the new Italian regulations (D.Lgs 2014/26), EU regulations (EU Directive 2010/63), and the ARRIVE guidelines.

### 4.4. Experimental Protocol

Cashew nuts (100 mg/kg body) were administered for 3 consecutive days through gavage. After the treatment schedule, the rats were fasted for 12 h. The induction of intestinal ischemia and reperfusion injury was performed as already described [34]. After anesthesia induction and midline laparotomy, ischemia and reperfusion injury were induced by clamping the superior mesenteric artery. After 30 min, the vascular clamp was removed allowing animals to 1 h of reperfusion. During the whole process, the body temperature of the rats was maintained at 37.5 °C, using a heating lamp. After this time, animals were sacrificed and tissues were harvested for histological and biochemical analyses. In another set of experiments, following reperfusion, the various groups of animals were observed for 4 h to evaluate survival differences [25].

### 4.5. Experimental Groups

Rats were randomly divided into the following groups:

(1) I/R + vehicle (saline): rats treated with vehicle and subjected to the surgical procedure described above;

(2) I/R + cashew nuts (100 mg/kg): rats treated with cashew nuts (100 mg/kg) and subjected to the surgical procedure described above;

(3) SHAM groups. animals were operated with surgical steps; however, they were not subjected to I/R and were treated with either vehicle or cashew nuts.

The tested dose was chosen based on previous studies performed in our laboratories [15,22].

In another set of experiments, we used melatonin as positive control (10 mg/Kg) [24,25,54].

### 4.6. Measurement of Lipid Peroxidation

The lipid peroxidation was evaluated by reaction between MDA (malondialdehyde), thiobarbituric acid, and lipid peroxides and measured spectrophotometrically at 532 nm [55]. Results were expressed as nanomole TBA (thiobarbituric acid) reactants formed per gram wet tissue.

### 4.7. Myeloperoxidase Activity

Myeloperoxidase (MPO) activity, an indicator of PMN accumulation, was determined spectrophotometrically at 650 nm [56].

### 4.8. Measurement of Protein Carbonyl Content

Protein carbonyl content (PCC) was evaluated spectrophotometrically at 370 nm by the reaction between 2, 4-Dinitrophenylhydrazine and carbonyl group [57]. The results were expressed as nanomoles of carbonyl per milligram of protein.

### 4.9. Determination of Antioxidant Enzyme Activities

Ileum tissues were homogenized, and the supernatants were collected for determining total glutathione (Glutathione Assay Kit; Trevigen Inc., Gaithersburg, MD, USA). The results of GSH levels were expressed as nanomole per milligram of protein [34]. SOD activity was determined on ileum tissue homogenate according to the principle of nitro blue tetrazolium reduction assay [58]. The superoxide dismutase (SOD) activity is expressed as U/mg of protein. The catalase (CAT) activity was estimated by decreased in absorbance of H_2_O_2_ at 240 nm [59]. The GPx was performed as described [34]. Oxidized glutathione (GSSG) is reduced by glutathione reductase and NADPH. The oxidation of NADPH to NADP + is evaluated by decrease in absorbance at 340 nm. GPx activity is expressed as U/mg of protein. The glutathione-S-transferase (GST) activity was determined spectrophotometrically at 340 nm [34]. 1 U = amount of enzyme producing 1 mmol of CDNB-GSH conjugate/min.

### 4.10. Evaluation of TNF-α, IL6, IL−1β, ALT, AST, and Creatinine Levels

The serum interleukins IL−1β, IL6, and TNF-α levels were determined using an ELISA kit (R&D System Inc., Minneapolis, MN, USA). Levels of glutamic oxaloacetic transaminase (AST), alanine transaminase (ALT), and creatinine were detected by using the commercial kits (Abcam, Milano, Italy).

### 4.11. Histological Examination

Ileum tissues were collected after 1 h of reperfusion. After fixing the tissues in buffered formaldehyde solution (10% in PBS), histological sections were stained with hematoxylin and eosin and evaluated using a Leica DM6 microscope (Leica Microsystems SpA, Milan, Italy) associated with Leica LAS X Navigator software (Leica Microsystems SpA, Milan, Italy). The morphological criteria were considered as already described [24]. All images were acquired at 10× magnification (250 µm).

### 4.12. Western Blot Analysis

Western blots were performed as described from our previous studies [60]. Specific primary antibody: anti-NRF2 (1:600, Santa Cruz Biotechnology) or anti-iNOS (1:700; Santa Cruz Biotechnology) or anti-IkB-α (1:700; Santa Cruz Biotechnology) or anti-NF-kB (1:700; Santa Cruz Biotechnology) was mixed in 1 × PBS, 5% *w/v* nonfat dried milk, and 0.1% Tween 20, and were incubated at 4 °C, overnight. Afterwards, blots were incubated with peroxidase-conjugated bovine antimouse IgG secondary antibody or peroxidase-conjugated goat antirabbit IgG (1:2000, Jackson Immuno Research) for 1 h at room temperature. To verify the equal amounts of protein, membranes were also incubated with the antibody against laminin (1:1000; Santa Cruz Biotechnology) and GADPH (1:1000; Santa Cruz Biotechnology). Signals were detected with enhanced chemiluminescence detection system reagent (Super-Signal West Pico Chemiluminescent Substrate, Pierce). The relative expression of the protein bands was quantified by densitometry with Bio-Rad ChemiDoc XRS software and standardized to β-actin levels. Images of blot signals were imported to analysis software (Image Quant TL, v2003).

### 4.13. Immunohistochemical Localization of Cell Adhesion Molecules (ICAM−1, P-Selectin), Poly(ADP-Ribose Polymerase) (PARP), and Nitrotyrosine

Immunohistochemical analysis was performed as already described [56]. The sections were incubated overnight with primary antibodies, i.e., anti-P-selectin antibody (Santa Cruz Biotechnology), anti-ICAM−1 antibody (Santa Cruz Biotechnology), antinitrotyrosine antibody (Millipore), and anti-PARP antibody (Santa Cruz Biotechnology). All sections were washed with PBS and then treated as previously reported [61]. Stained sections from each mouse were scored in a blinded fashion and observed using a Leica DM6 microscope (Leica Microsystems SpA, Milan, Italy) following a typical procedure [22]. The histogram profile is related to the positive pixel intensity value obtained [15]. All images were acquired at 10 × magnification (250 µm).

### 4.14. Measurement of Intestinal Permeability

Animals from all groups were starved for 4 h and subsequently administered with fluorescein isothiocyanate (FITC)-dextran (600 mg/kg). After 4 h, rats were sacrificed by cardiac puncture. Serum FD−40 levels were detected by fluorometry [62].

### 4.15. Bacterial Translocation

The mesenteric lymph nodes (MLN) and the caudal lymph nodes (CLN) were harvested and investigated for bacteriological analysis [63].

### 4.16. Statistical Evaluation

All values in the figures and text are expressed as mean ± standard error of the mean (SEM) of N number of animals. Results were analyzed by the *t*-test. A *p*-value < 0.05 was considered significant. * *p* < 0.05 vs. sham, # *p* < 0.05 vs. vehicle, ** *p* < 0.01 vs. sham, ## *p* < 0.01 vs. vehicle, *** *p* < 0.001 vs. sham, ### *p* < 0.001 vs. vehicle.

## Figures and Tables

**Table 1:** Nutritional profile of cashew kernel samples from Ivory Coast. Results are expressed for 100 g of cashew kernel samples.

Nutrients	Units	Cashew Kernel
Ash	g	2.68 ± 0.12
Dietary fiber (total)	g	4.48 ± 0.27
Lipids (total)	g	44.19 ± 1.85
Moisture	g	5.40 ± 0.28
Protein	g	22.46 ± 1.05
Sugars (total)	g	30.95 ± 1.44
Total phenols	mg	80.01 ± 2.65

## Supplementary Materials

Figure S1. Effect of cashew nuts on I/R shock-induced intestinal damage.
