# Supplementary material for: Consumption of Anacardium occidentale L. (Cashew Nuts) Inhibits Oxidative Stress through Modulation of the Nrf2/HO−1 and NF-kB Pathways
Source: Molecules. 2020 Sep 26;25(19):4426. doi: 10.3390/molecules25194426 (PMC7582295; doi:10.3390/molecules25194426)
Supplement: Supplementary file 1 [file molecules-25-04426-s001.pdf]

Figure 1S. Effect of cashew nuts on I/R shock induced intestine damage: H&E staining: Sham (A), Vehicle (B), Melatonin (C), Cashew nuts (D), Histological injury score (E), MPO activity (F),Lipid Peroxidation (G), CAT (H), SOD (I). Results were analyzed by the t-test. A p-value <0.05 was considered significant. * $p < 0.05$ vs. sham, # $p < 0.05$ vs. vehicle, ** $p < 0.01$ vs. sham, ## $p < 0.01$ vs. vehicle, *** $p < 0.001$ vs. sham, ### $p < 0.001$ vs. vehicle.

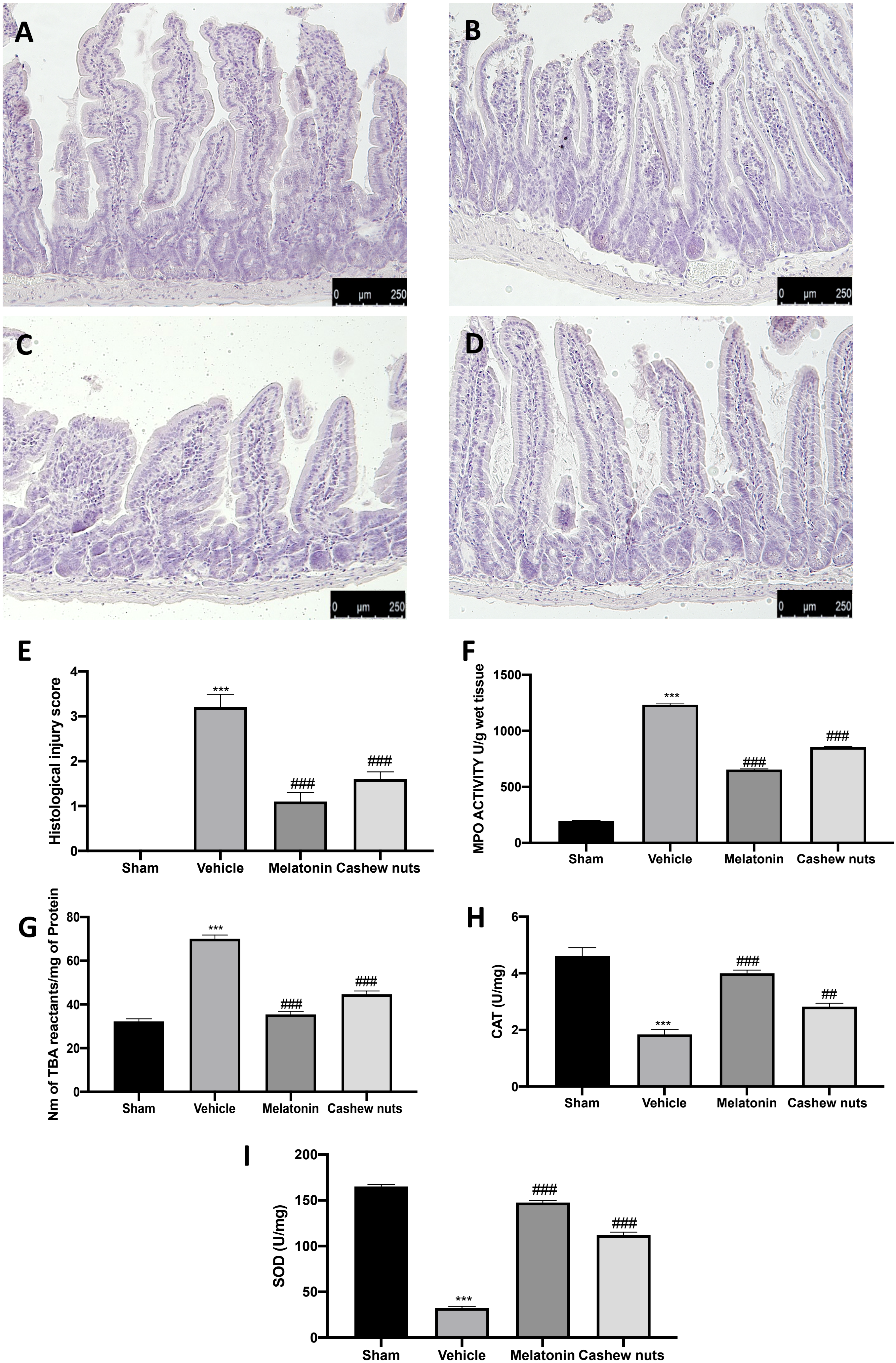

A
0 µm 250
B
0 µm 250
C
0 µm 250
D
0 µm 250
E
Histological injury score
0
1
2
3
4
***
###
###
Sham
Vehicle
Melatonin
Cashew nuts
F
MPO ACTIVITY U/g wet tissue
0
500
1000
1500
***
###
###
Sham
Vehicle
Melatonin
Cashew nuts
G
Nm of TBA reactants/mg of Protein
0
20
40
60
80
***
###
###
Sham
Vehicle
Melatonin
Cashew nuts
H
CAT (U/mg)
0
2
4
6
***
###
##
Sham
Vehicle
Melatonin
Cashew nuts
I
SOD (U/mg)
0
50
100
150
200
***
###
###
Sham
Vehicle
Melatonin
Cashew nuts
